# The hepatic transcriptome in human liver disease

**DOI:** 10.1186/1476-5926-5-6

**Published:** 2006-11-07

**Authors:** Nicholas A Shackel, Devanshi Seth, Paul S Haber, Mark D Gorrell, Geoffrey W McCaughan

**Affiliations:** 1 AW Morrow Gastroenterology and Liver Centre, Centenary Institute of Cancer Medicine and Cell Biology, Royal Prince Alfred Hospital and The University of Sydney, Sydney, Australia

## Abstract

The transcriptome is the mRNA transcript pool in a cell, organ or tissue with the liver transcriptome being amongst the most complex of any organ. Functional genomics methodologies are now being widely utilized to study transcriptomes including the hepatic transcriptome. This review outlines commonly used methods of transcriptome analysis, especially gene array analysis, focusing on publications utilizing these methods to understand human liver disease. Additionally, we have outlined the relationship between transcript and protein expressions as well as summarizing what is known about the variability of the transcriptome in non-diseased liver tissue. The approaches covered include gene array analysis, serial analysis of gene expression, subtractive hybridization and differential display. The discussion focuses on primate whole organ studies and in-vitro cell culture systems utilized. It is now clear that there are a vast number research opportunities for transcriptome analysis of human liver disease as we attempt to better understand both non-diseased and disease hepatic mRNA expression. We conclude that hepatic transcriptome analysis has already made significant contributions to the understanding of human liver pathobiology.

## Background

The sequencing of the human and other genomes has heralded the age of functional genomics. Although an invaluable resource in understanding human biology and disease, the frequent lack of sequence correlation with a defined tissue or disease phenotype has led to the genomic sequence databases being huge reservoirs of knowledge that mostly aid but do not direct research. We have the start of the map for human disease but only limited understanding of how it unfolds. Moreover, this genomic "gene map" is invariant across an entire organism and it is the expression of messenger RNA (mRNA) gene transcripts and resultant protein expression that defines normal molecular homeostasis and pathobiology. Functional genomics studies attempt to correlate gene mRNA transcript expression with a characterised phenotype thereby inferring function.

The entire mRNA transcript pool within a cell or tissue has been labelled the transcriptome [1-3]. Similarly, the proteome refers to the entire protein pool. Understanding the regulation and expression of transcriptomes or proteomes in a disease specific context is pivotal to understanding human disease. Further, although proteins are the mediators of molecular pathobiology proteome expression is ultimately controlled by the transcriptome. Approaches aimed at understanding the relationship between mRNA and protein expression are complementary and important in understanding disease [1,2]. No single approach or methodology to examine the transcriptome is "best" or "correct" and one of the central goals of this review is to highlight the benefits and deficiencies of many current approaches being utilized to examine transciptomes (Table 1). Additionally, understanding the relationship between the transcriptome and proteome is essential in interpreting functional genomic studies.

**Table 1:** Transcriptome analysis.

***Open systems***	**Advantages**	**Disadvantages**	**Comments**
**Representational differences analysis (RDA)**	• Sensitive method • Sequence data obtained • Alternately spliced transcripts can be easily identified	• "Hit and miss" approach • Not suitable for transcriptome profiling • Sequencing intensive • Transcript representation may change and results need to be verified	• Typically used for identification of novel differentially expressed transcripts • Most commonly used variant of this method is Suppression Subtractive Hybridization (SSH)
**Differential display (DD)**	• Sensitive method • Sequence data obtained	• High false positive rate • Not suitable for transcriptome profiling • Transcript representation may change and results need to be verified	• Not currently a widely favoured methodology
**Serial analysis of gene expression (SAGE)**	• Transciptome profiling possible • Transcript representation retained	• Limited sequence data obtained • Sequencing intensive method • Often fails to account for transcript alternate splicing	• SAGE suitability for transciptome profiling is reliant on extensive sequencing

***Closed Systems***	**Advantages**	**Disadvantages**	**Comments**

**Gene arrays**	• Characterized target sequences on the arrays • Extremely small feature size • Very high through put methodology	• Restricted gene pool that may sample rather than profile the transcriptome • Variability • Signal amplification often needed for biopsies • Often fails to account for transcript alternate splicing • Data generated can be a bioinformatics challenge • Inconsistencies with analysis approaches	• Preferred transciptome profiling method • Gene alternate splicing can be addressed by using "tilling arrays" • MIAME is designed to overcome methodological inconsistencies

Organ specific research has lagged behind the understanding of general biological processes. However, most human disease is defined by unique changes to organ specific transciptomes and proteomes. Further, the transcriptome and proteome of individual cells is defined by the intracellular milieu within an organ [3]. Therefore, understanding the genome expression in an organ specific context is pivotal to understanding normal homeostasis and pathobiology. The normal variation, age related changes and sex differences in organ related gene expression are further important aspects, as well as frequent confounding variables, in understanding and interpreting changes in organ transcriptome expression. The situation in diseased tissue is even less well understood, examples from non-liver tissue has shown that transcriptomes can dramatically increase in complexity with disease [4]. Reducing the complexity of organ transcriptomes by studying individual cell types is a reductionist approach to understanding gene expression [3]. However, many forms of adult liver disease have no suitable in-vitro models of disease and the study of the whole liver is presently the principle means of understanding disease pathogenesis.

The study of intrahepatic gene expression has been greatly enhanced by functional genomic methodologies. However, the examination of the normal hepatic transcriptome has received limited attention. An overall understanding of hepatic transcriptome expression is being unravelled as a by-product of focused attempts at understanding individual liver disease phenotypes. Questions about the complexity of the hepatic transcriptome, including individual variability and extent of change in liver disease are poorly understood. One of the focuses of this review is to outline the known complexity of the hepatic transcriptome in normal homeostasis and disease. Disease specific examples will be used to highlight progress made due to use of functional genomic methodologies as well as highlighting strengths and weaknesses of the techniques used to study transcriptomes.

## The liver transcriptome

There are estimated to be in excess of 32 000 protein encoding genes in the human genome. Further, there are an unknown number of functionally significant alternately spliced transcripts arising from these genes that may exceed 100 000. How many of these transcripts are expressed in the liver is unknown. Resources for identifying and comparing organ transcriptomes are rare. One method of inferring complexity is to examine GenBank human UniGene clusters of non-redundant gene sets [5]. These UniGene clusters are compiled from mRNA and expressed sequence tags (EST) and as a group represent a species' transcriptome [5]. Currently the human UniGene assembly of clusters (Build 180) has over 5 million sequences representing 52 888 non-redundant transcripts. Parsing key word searches approximately 26% of transcripts (representing 13 627 clusters) were identified in liver tissue, this compares to brain (46%), lung (40%), kidney (35%), colon (32%) and heart (23%); Parsing string used ("liver" or "hepatic") and "human" for UniGene Build 180. Coulouarn et. al. used a similar approach and identified 12 638 non-redundant clusters from liver tissue (UniGene Build 129) [6]. Further, Serial Analysis of Gene Expression (SAGE) libraries can also provide some insight into the complexity of the liver transcriptome [7,8]. Two SAGE libraries from normal human liver identified 15 496 and 18 081 unique transcripts from a total number of 66 308 and 125 700 tags respectively [7,8]. In a SAGE comparison of multiple organs 32 131 unique tags were identified (from a total of 455 325 tags) of these 56% were expressed in the liver compared to brain (75%), breast (81%) and colon (91%) [8]. Therefore it is clear that the normal liver has a complex transcriptome expressing many thousands of transcripts. Interestingly, the SAGE comparison of gene transcripts from various tissues has identified organ related chromosomal domains such as 6p12.1 associated with hepatic xenobiotic metabolism [8].

Microarray analysis of normal human liver by Yano et. al. highlights many of the problems in examining the non-diseased liver transcriptome [9]. A total of 2 418 genes were examined in 5 normal patients with only 50% of these transcripts being detected in 4 out of five patients. Further only 27% of genes had co-ordinate expression in these normal patients. Therefore, in addition to the liver having a complex transcriptome there appears to be significant individual variability in transcript expression. This is further highlighted by the observation of Enard et. al. that duplicate liver samples from the same individual differed by 12% (technical variation) but that intraspecies variation was as pronounced as interspecies variation in hepatic mRNA transcript expression comparing chimpanzees and humans [10].

The situation in liver disease is even more poorly understood. Transcriptomes, especially those undergoing malignant transformation can double or even triple in complexity. The liver appears to be similar in this regard to other transcriptomes. In a SAGE analysis of intrahepatic hepatitis C virus (HCV) infection the number of unique transcripts identified increased by 18% to over 55% of the total of normal liver transcripts in HCV and HCV with hepatocellular carcinoma (HCC) respectively [11]. Additional SAGE expression data demonstrates that the complexity of the liver transcriptome can increase by 2 to 3 fold in the transition from normal liver to cholangiocarcinoma. Therefore, are comparisons of transcriptomes, even from the same organ, which differ so dramatically in complexity valid? These problems are highlighted in a number of functional genomic studies. The "Liverpool" nylon array examination of inflammation associated liver disease (patients with hepatic abscesses, metastases and cholangitis) from a total of 9 858 genes identified 154 genes associated with an inflammatory acute phase (AP) response [6]. However, this study used a liver gene set derived from a human UniGene assembly that predominantly includes those transcripts that are only expressed in non-diseased liver or hepatic neoplasia. Therefore, this gene array may not detect transcripts involved in intrahepatic inflammation if they were not previously identified in liver tissue. Further, 59% of the genes that appeared to associate with an acute phase response were eliminated from the analysis as they did not encode for a known inflammation associated function [6]. The possibility that these genes encode for previously unrecognised inflammation associated transcripts was not investigated. Additionally, genes known to be associated with an AP response were not present in all individuals with liver inflammation. Moreover this study identified only 880 genes (8.9%) that had liver restricted expression [6]. This study highlights disease and technical discrepancies that need to be considered in functional genomic studies.

### Relationship between mRNA and protein expression

Proteins rather than mRNA are the major effectors of cellular and tissue function. The study of mRNA expression assumes that changes in mRNA expression reflect changes in protein expression. There are many examples, such as post-translational modifications, where protein expression or function is not controlled by mRNA expression [12,13]. In the intact non-diseased liver tissue, approximately 25% of the changes in the mRNA transcript expression are not accompanied by changes in the expression of the corresponding protein. Studies comparing mRNA and protein expression in liver are few. Anderson et. al. showed a poor correlation of the liver tissue abundance of 19 proteins and corresponding mRNA transcripts (correlation coefficient of only 0.48). Further, they isolated 50 abundant mRNA transcripts of which 29 encoded secreted proteins [14]. However, of the 50 most abundant proteins they isolated none were secreted [14]. There is a bias in mRNA pools, when compared to protein expression, towards an over-representation of both secreted protein transcripts and high abundance mRNA transcripts, such as glyceraldehyde-3-phosphate dehydrogenase, has been repeatedly demonstrated [14-17]. Human proteome and transcriptome comparisons are rare and complicated by our heterogenous multi-organ structure. In prototype organisms (yeast *Saccharomyces cerevisiae*) comparison of the proteome and transcriptome showed a correlation of 0.94 for all proteins and genes but a correlation of only 0.36 for lowly expressed proteins and genes [18]. The same study went on to show that mRNA expression varied up-to 20 fold with constant protein expression and that the protein expression could differ by greater than 30 fold when mRNA expression remained constant [18]. However, another study in *Saccharomyces cerevisiae*, showed a good correlation (r = 0.76) between lowly expressed proteins and mRNA expression with an estimated mRNA abundance as low as 10 transcripts per cell [19]. These two studies do shed light on some of the pitfalls inherent in human transcriptome analysis. Specifically, the importance of estimating the frequency of mRNA transcript and protein expression within homogenous (i.e. cell lines) compared to heterogenous (i.e. organs) cell populations.

A prototype human cell such as a lymphoblast has a mass of 670 pg, contains 67 pg of protein (a total of 10^9 ^polypeptide molecules representing 4,000 different proteins) and 0.024 pg of mRNA (4 × 10^4 ^transcripts representing 5,000 different mRNA) [20]. Rare mRNA transcripts present at a frequency of one copy per cell may well be missed based on the cell population frequency and size of the mRNA pool being analysed [20]. This is an important consideration for hepatologists utilizing biopsy specimens where the non-parenchymal cell subpopulation abundance is low and subject to sampling error [21-23]. Additionally, it is apparent that if mRNA amplification is utilized in functional genomic experiments then every effort needs to be made to ensure gene, especially low abundance gene, representation is retained. Further, in functional genomic methods that "sample" the transcriptome, such as serial analysis of gene expression (SAGE), differential display and subtractive hybridisation many low abundance transcripts will be missed without sampling of a very large numbers of clones identified. Therefore, most of these "sampling" methods are used as a means of identifying differential expressed mRNA rather than profiling a transcriptome. Detection of protein expression differs as the analytically threshold is the limiting factor. Currently proteomic detection requires picomole amounts of protein (10^12 ^– 10^13 ^peptides) [20]. In a homogenous cell population 90% of the cellular protein mass is due to the 100 most abundant proteins and a further 1200 proteins account for another 7% of the protein mass that is detectable on typical proteomic analysis (from a lysate of 10^6 ^cells) [20]. However, the remaining 3% of the protein mass includes 2800 proteins (over 50% of the different protein species) that fall below the threshold of detection for typical proteomic analysis [20]. Therefore, differential protein expression or proteomics can document changes in protein expression but is currently restricted to moderate to high abundance proteins as well as being technically demanding. Ultimately, the protein expression in every cell is controlled by the transcriptome although the relationship between individual gene transcripts and the corresponding protein expression may not at first, be clear.

## Functional genomics

It has been widely asserted that; "We know the sequence so now we need to understand the function". This has led to a plethora of new high through-put functional genomics methodologies directed at correlating gene expression with defined tissue or disease phenotypes. Most commonly mRNA transcript expression is compared in various states using techniques such as microarray analysis, differentially display (DD), subtractive hybridisation (SH) and SAGE. However, functional genomics approaches are not confined to examining transcriptomes as there are a range of methodologies that can examine proteomes. Further, the individual variability in gene expression and function due to sequence differences (nucleotide polymorphisms) is now being widely correlated with disease phenotypes. The distinguishing feature of all of these functional genomic methodologies is their high through-put nature and generation of large data sets. These techniques are confounded by problems with reproducibility and difficulties in interpretation. Indeed the correct method of analysis of these massive data sets is another new area of intense research efforts.

Functional genomic methodologies can be divided into two broad groups; (A) methods that require pre-existing knowledge of the gene sequence "closed architecture systems" or (B) methods that require no a priori knowledge of gene sequence "open architecture systems" (Table 1). Open architecture systems excel at finding novel sequence differences such as unrecognized splice variations but suffer from requiring sequencing intensive methodologies and frequently yielding results that are descriptive rather than truly quantitative. This has led to the extensive use of supplemental methodology to confirm and validate gene expression identified by open architecture systems. Further in the absence of immense sequencing efforts open architecture systems tend to "sample" the transcriptome rather than profiling the entire transcriptome. In contrast closed architecture systems enable a consistent comparison of gene pools in various disease states but suffer from an inability to detect novel sequence differences. However, with the sequencing of the human genome the number of potentially novel genes that open architecture system can study compared to closed architecture system has decreased.

## Gene array analysis

Array analysis has become the preferred means of rapidly determining differential gene expression in hundreds to thousands of mRNA transcripts in a single experiment [3,24-27]. From its inception in the 1990's the proliferation of this methodology has seen a number of different techniques arise [3,24-27]. However, the underlying principle of all array experiments remains the same with the application of DNA representing individual genes onto a substrate that is investigated with a sample labelled to enable detection following hybridisation. The principal differences in array methodologies relate to the samples applied to the array, the substrate the samples are fixed to and the method of preparing the probe that is subsequently hybridised to the array. The substrate the DNA is fixed to divides the array techniques into those based on membranes (typically nylon but now also plastic), glass arrays and chip arrays. This approximates the density with which the DNA is represented on the array, with the membrane based arrays having the lowest density of gene targets through to the gene chip arrays where there is an extremely high density of DNA targets on each chip.

Starting material for array analysis is often limited requiring the use of amplification technologies [3,34]. Three technologies are readily available to amplify mRNA from even a single cell. Eberwine amplification is a linear amplification method that uses a T7 RNA polymerase to produce amplified RNA (aRNA) [35,36]. The poly(A) PCR method is a PCR based method that is biased towards small, 100 to 500 bp PCR products [37]. The final method is SMART cDNA amplification (Clontech, CA, USA) which is also a PCR based method that preferentially amplifies full length cDNA [38-43]. All three amplification methods have been successfully used to amplify liver tissue for gene array analysis. Importantly, all three methods have been shown to retain the representation of transcripts from the starting RNA pool.

Data analysis of array results is a significant bio-informatics challenge. Ascertaining the significance of individual gene differential expression should be determined for each set of experimental conditions. Gene expression from array experiments is typically presented as ratios of differential expression in compared disease states [44]. The level of significant differential expression used by most investigators is a 2.0 fold increase or decrease but differences as low as 1.4 fold have been demonstrated to be significant [45]. Threshold ratios of differential gene expression are designed to distinguish "array noise" from "biological noise" [44]. The variation of an individual gene on a single array is estimated to be between 8 to 18% with the array-to-array difference of 15% [44]. However, the animal-to-animal coefficient of variation is estimated at 18 to 60% [44]. Additional phenotype differences that can have profound effects on hepatic transcriptome expression include circadian changes [46-48], age [49] and diet [50-52]. Sophisticated means of analysis now enable the researcher to perform covariate analysis (a covariate is a variable that contains contextual information for a sample or gene) [44,53]. For instance, in a study of colorectal carcinoma cell lines the expression of dihydropyrimidine dehydrogenase (DPYD) negatively correlated with sensitivity to 5-fluoruracil (FU) [54]. This is a covariate correlation that makes physiological sense, as DPYD is the rate-limiting enzyme degrading 5-FU [54].

Linking the array expression data with pathogenic process is performed with or without a priori knowledge of gene function (also known as supervised and unsupervised methods respectively) [3,55-60]. Grouping or clustering of genes to reduce the complexity of the data set is the principle method of array analysis used [3,60,61]. In supervised methods, genes included on an array are grouped with pathogenic processes and pathways according to known gene function [3,60]. An alternative supervised approach clusters known diseases states together and then ascertains in which disease group an uncharacterised sample belongs based on the analysis of gene expression. Unsupervised analysis is a powerful approach that makes no assumptions about the function of a gene and clusters genes based on their similarity in pattern of expression [57,60]. Hierarchical pair-wise clustering is the most common means now employed of grouping genes without a priori knowledge of function [57,60-62]. Alternative approaches include self-organising maps, Bayesian clustering, k-means clustering and neural networks [57,60,63]. Essentially, all of these approaches aim to "extract order from chaos" by "grouping and feature reduction" [63]. Importantly, these analyses are reliant on the use of multiple disease comparisons. Once a group of genes has been identified (typically as a list of accession numbers) a number of resources are available to rapidly identify important pathogenic pathways. These resources include both commercial computing packages and Internet based services [3].

### Gene array analysis publications

Array analysis has become a commonly published methodology. Microarray analysis leads the way with more published experiments than other forms of array experiments. Array experiments offer "...a whole new way of looking at cellular connections" [44]. Array analysis has targeted identification of "candidate" genes in pathobiology, identification of gene networks, characterisation of gene ontologies and phylogeny as well as the classification of pathobiology [44]. The diagnostic potential of array analysis is further suggested by two early pivotal studies conducted in human leukemia and lymphoma patients. Golub and colleagues were able to distinguish acute myeloid leukemia from acute lymphoid leukemia (ALL) based on the gene expression pattern on microarrays [64]. Further, they were able to identify important "class predictor" sets of genes and were further able to divide ALL samples into those of B- or T-cell origin [64]. The microarray analysis of Alizadeh et. al. showed that diffuse large B-cell lymphoma could be divided into two broad groups based on the pattern of gene expression consistent with either a germinal centre origin or a peripheral B-cell origin [65]. Further, patients with the peripheral B-cell origin gene profile had a significantly greater long-term survival [65]. Following these initial studies there are now a number of manuscripts that correlate clinical outcome in malignancy with tumor mRNA transcript expression. In a number of studies microarray profiling of transcript expression was the best predictor of clinical outcome. Therefore, gene expression profiles are now moving from the research laboratory into clinical practice as a diagnostic and prognostic tool in malignancy. However there are very few studies correlating clinical outcome with gene expression in non-malignant disease. This is consistent with diseases in which there is a marked difference in gene expression (i.e. in malignancy) being the easiest to discriminate by gene array analysis. This underscores the need to understand normal variability and to address the issue of reproducibility if gene array analysis is to be used for predicting outcome in non-malignant disease.

### Gene array analysis in human liver disease

Array analysis in human liver disease has now profiled the intrahepatic gene expression in non-diseased liver, a number of diseases and animal models of disease. Presently there are in excess of 200 published gene array studies of human liver disease or studies that utilize human liver tissue (Table 2). This represents roughly 40% of the greater than 500 published papers utilizing gene arrays to study liver pathobiology. Most of these studies attempt to understand pathobiology by examining mRNA transcript expression. There are few publications in human liver disease where gene expression is correlated with clinical outcome.

**Table 2:** Summary of study findings following transcriptome analyses of human liver disease.

**Pathology**	**Principal Findings**	**Reference**
Normal liver	Marked variation in normal liver transcript expression	[6, 7, 9]
	Innate immune gene induction with the acute phase response	[6]
Hepatitis C (HCV)	Innate immune activation with acute HCV infection	[68, 71]
	Immune induction with chronic HCV infection	[67, 68, 69, 71, 75, 78]
	NS3/4A induction of innate immune responses	[73]
	NS5A induced NF-kappaβ activation	[170]
	Upregulation of specific IFN response genes predicts treatment response	[74]
	Gene expression associated with HCV HCC	[11, 79, 151, 171]
	Immune activation with HCV recurrence post transplantation	[86, 87]
	Immune response to chronic HBV infection	[69, 75]
Autoimmune hepatitis (AIH)	Intrahepatic transcript expression in AIH cirrhosis	[76, 82]
Alcohol associated liver disease (ALD)	Intrahepatic transcript expression in alcohol liver injury	[89]
Non-alcoholic fatty liver disease (NAFLD)	Gene expression in hepatic steatosis	[92–94], [93]**
	Intrahepatic transcript expression in non-alcoholic steatohepatitis (NASH)	[92]
Biliary liver disease	Intrahepatic transcript expression in primary biliary cirrhosis	[81, 82]
	Intrahepatic transcript expression in primary sclerosing cholangitis-related cirrhosis	[81]
	Transcript expression distinguishing embryonic and perinatal forms of biliary atresia	[95]
Hepatocellular Carcinoma (HCC)	Novel gene expression (possible tumour markers) in HCC	[80, 83]
	Transcript expression in viral hepatitis associated HCC	[172–175]
	Transcript expression in metastatic HCC development	[176, 177]
	Transcript expression in associated with prognosis and/or recurrence of HCC	[178, 179]
Cholangiocarcinoma	Transcript expression of intrahepatic cholangiocarcinoma	[180]

**Combination of transcriptome and proteome analysis.

### Gene array analysis in human liver disease – Viral hepatitis

Gene array analysis has provided novel insights into the pathogenesis of hepatitis B (HBV) and hepatitis C (HCV) liver injury. Importantly, both of these diseases have been extensively studied using primate models of infection or in human disease. Further, examination of HCV liver injury is restricted to studies in humans and chimpanzees as there are no in-vivo animal models of infection. The insights into viral hepatitis pathogenesis offered by gene array analysis has shown a number of common aspects such as the interferon (IFN) associated gene response as well as distinct differences that are helping us to understand the unique intrahepatic transcript "signature" of each viral infection. Additionally, studies of temporal changes in transcript expression are central to understanding clearance of virus, carrier states and injury in chronic persistent infection.

Acute HBV and HCV infection has been analysed in the chimpanzee [66-68]. Acute HBV infection is characterised by an absence of intrahepatic differential gene expression during HBV infection and the initial phase of virus replication. This is followed by the differential gene expression associated with T-cell receptor and antigen presentation. Subsequently gene expression associated with T cell recruitment (chemokines), T cell effector function (granzymes) and monocyte activation was observed. A later phase of clearance was associated with the expression of B cell related genes. This is in direct contrast to HCV infection and suggests that HBV infection fails to induce a significant innate immune response by acting in the initial phase of infection as a "stealth virus" [66,69]. Presently there are no published studies of acute or chronic HBV infection in humans. Our own studies of cirrhotic HBV explants demonstrated less differential gene expression than studies comparing normal liver and HCV associated cirrhosis. Chronic HBV infection is characterised by intrahepatic upregulation of stress response, cell cycle and immune response associated genes. This is similar to findings reported by others (personal communications).

Chimpanzee models of HCV have helped delineate the development of the intrahepatic immune response in acute and chronic HCV infection. Studies of chimpanzees during acute HCV infection which showed a dramatic intrahepatic response with an increase in IFN response genes as early as two weeks post infection [67,68]. The chimpanzee studies demonstrated an initial response with elevated IFN-alpha/beta associated with a biphasic intrahepatic immune response to HCV resulting in viral clearance [67,68,70]. The IFN induced genes such as ISG15 and ISG16-jun were initially strongly upregulated followed by over 50 fold induction of Th1 associated transcripts such as MIG (CXCL9) and IP10 (CXCL10). Subsequent increased CXCL-10 (IFN-induced protein 10 (IP-10)) and midkine (MK) peaking at 6 weeks is consistent with an adaptive, Th1 associated, immune response clearing infected hepatocytes of the virus [68].

Comparison of chimpanzees that cleared acute HCV infection compared to an animal that had virus persistence has provided further insight into the balance between viral clearance and peristance [71]. In these experiments, Su et al. [71] observed upregulation of genes associated with the early response (which correlated with viral load) including many IFN alpha induced genes; STAT 1, 2'-5' oligoadenylate synthetase, Mx1, ISP15 and p27. Interestingly, there was the induction of lipid pathway genes such as fatty acid synthetase, sterol response element binding protein (SREBP), down regulation of PPARα as well as hepatic lipase C and flotillin 2. The lipid pathway genes are associated with viral replication and studies using in vitro replicon experiments has demonstrated altered viral replication [72]. Further, the reduction in PPARα would be expected to be associated with insulin resistance, a feature of chronic HCV, but prior to this it was not an expected aspect of acute HCV infection. As noted previously [68] clearance of HCV was associated with the late induction of Th1 transcripts such as CXCL9 and CXCL10, MHC expression and T cell molecules such as CD8 and granzyme A. The induction of IFN alpha induced genes early in infection was also observed by Bigger et al but the timing did not correlate with clearance as high levels of these transcripts continued in the animal with viral persistence. This has also been seen in human studies. Further, functional studies in HCV replicon systems has shown that the NS3/4a was able to inhibit interferon alpha antiviral effector function by blocking the phosphorylation of IRF-3 a key protein in the antiviral response [73]. Therefore, chronic HCV infection induces a persistent intrahepatic IFN alpha antiviral response but the virus itself escapes this response via inhibition of the effector arm. However, microarray studies of the intrahepatic IFN alpha induced gene response show that this is variable and observed to be higher in patients not responding to pegylated IFN and ribavirin therapy consistent with resistance of the effector arm of the immune response to amplification by exogenous therapy [74]. In contrast, patients who had a sustained viral response (SVR) to pegylated IFN therapy had a lower expression of IFN genes consistent with amplification of the effector arm of the immune response by exogenous therapy resulting in viral clearance.

Chronic HCV infection has been studied in a number of ways using gene array analysis. The study of Honda et al. profiled gene expression in liver biopsy material in individuals with chronic hepatitis B (HBV) and chronic HCV comparing them to a single non-diseased control [75]. The authors concluded that chronic HCV infection was associated with a predominant anti-inflammatory, pro-proliferative, anti-apoptotic intrahepatic gene profile [75]. However, the presented data demonstrated widespread upregulation of pro-inflammatory genes such as IL-2 Receptor, CD69, CD44, IFN gamma inducible protein, MHC Class 1 genes and monokine induced by gamma IFN. These findings were similar to another study of HCV cirrhosis in which a pro-inflammatory Th1 associated transcript expression predominated [76]. The Th1 immune response is thought to be responsible for the accelerated fibrogenesis of HCV liver injury [77]. Fibrosis associated gene expression in HCV associated fibrosis has included upregulation of a wide variety of genes including PDGF, TGF-beta 3 [78]. Additionally, gene array analysis has identified potential novel mediators of HCV associated injury such as Frizzled related proteins, discoid domain related protein1 (DDR1), EMMPRIN and SARP-3 [76].

The premaligant potential of intrahepatic HCV infection has been studied by our group by comparing HCV cirrhosis with and without HCC by gene array analysis [79]. The upregulation of many oncogenes (i.e. TEL oncogene), immune genes (IFN gamma associated), fibrosis genes (integrins) as well as cell signalling (G coupled receptor kinase) and proliferation associated genes (cyclin K) was demonstrated in cirrhosis complicated by HCC. This is consistent with a premalignant cirrhotic response in HCV infection. Further, the data suggest that there is more cellular proliferation, immune activation and fibrosis in the liver of patients with HCC than those with cirrhosis alone. A key area of future research will be to ascertain wether such a profile can be recognised before HCC develops. This approach has a direct clinical application in identifying and screening high-risk patients.

Gene array studies of HCV infection has revealed new insights into the development of HCC in HCV, structural analysis of the HCV RNA genome and identified novel markers of HCV intrahepatic injury and HCV associated HCC. The study of Smith et al. utilized 13 600 gene microarrays to profile patients with HCV cirrhosis, HCV and HCC and normal liver [80]. The results identified 87 upregulated and 45 down-regulated genes that appear to be markers of HCV liver injury [80]. Importantly, the analysis aimed to exclude genes expressed in normal liver, other forms of cirrhosis or HCC. Genes such as ILxR (IL-13 receptor a2), CCR4 and cartilage glycoprotein 39 (GP-39) were identified [80]. However, the study of Smith et al. highlights the problems with the interpretation of these large data sets using small numbers of patient samples; does the identified gene expression represent unique disease or phenotype associated gene expression or the stochastic probability of identifying a small cohort of genes from the many thousands being analysed? Cleary studies such as these, as powerful as they are, need to be validated by alternative methodologies in large patient groups. Our own approach to validation has been to confirm important gene expression identified in these studies by real-time RT-PCR in a larger cohort of patients [78,81]. Indeed our own results have confirmed the increased expression of GP-39 in HCV associated cirrhosis identified following subtractive hybridization and confirmed this finding by real-time RT-PCR [82].

Hepatocellular carcinoma proliferation in HCV associated liver injury has been studied by array analysis. This has resulted in a plethora of potentially novel tumour markers being identified. These include the serine/threonine kinase 15 (STK15) and phospholipase A2 (PLA2G13 and PLA2G7) that were shown to be increased in over half of the tumours identified [80]. However, a different study implicated different gene groups in HCV associated HCC; cytoplasmic dynein light chain, hepatoma derived growth factor, ribosomal protein L6, TR3 orphan receptor and c-myc [83]. The clustering analysis in this study showed that the expression of 22 genes in HCC related to differentiation of the malignancy with over half of these genes being transcription factors or related to cell development or differentiation [83]. Although many of these genes can be implicated in HCC development they often identified in large gene sets in end stage disease. Therefore, whether these genes represent cause or effect is unknown. Additionally, the number of differing gene sets being examined by the gene arrays being utilized is almost as great as the number of studies using them. Further as these gene sets still only represent a fraction of the transcriptome being examined they selectively identify differentially expressed genes. The same group went on to examine the expression of genes in hepatocyte cell lines expressing α-fetoprotein (α-FP) [84]. Comparison with non α-FP producing cell lines showed that the hepatocyte lines had a similar pattern of gene expression [84]. Further, the cell lines Huh-7, Hep3B and HepG2 clustered together suggesting that these cells lines are closely related [84]. Indeed these cell lines shared 254 genes (out of 930) that were commonly up-regulated [84]. Genes increased in the α-FP producing cell lines included α-FP, ephrin-A1, TGF-α2, MMP-2 and IGF-II [84]. This is entirely consistent with the known role of these genes in carcinogenesis and more specifically with transcriptional regulation of MMP-2 by IGF-II [85]. The oncogenic potential of viral components, especially core protein, has been implicated by transcriptome profiling in the pathogenesis of HCV associated HCC.

Gene array analysis of HCV recurrence in transplant allografts has provided novel insights into the molecular mechanisms of viral recurrence [86,87]. HCV recurrence in the liver graft is associated with expression of IFN-γ associated genes such as CXCL10 (IP-10), CXCL9 (HuMIG) and RANTES [87]. Further, antiviral IFN-α associated gene expression is seen in chronic HCV recurrence and during acute rejection associated with HCV recurrence [87]. Additionally, upregulation of NF-kappa β pathway during acute rejection in association with HCV recurrence appears to alter cellular apoptosis via changes in the expression of TRIAL associated genes [87]. Importantly chronic HCV recurrence in grafts is associated with Th1 associated gene expression similar to that seen in chronically HCV infected individuals that have not been transplanted [87]. In contrast, cholestatic HCV recurrence, which follows an aggressive course, is associated with a Th2 cytokine profile [87]. This suggests that the Th1 immune response suppresses viral replication whilst being profibrogenic [78,87,88]. In cholestatic HCV recurrence the unchecked viral replication is directly fibrogenic [87,88].

### Gene array analysis in human liver disease – alcoholic and steatotic liver disease

Alcoholic liver injury is an example of both a classical and atypical hepatotoxin. Microarray analysis has been applied to studies of neural tissue in an attempt to understand ethanol addiction. Intrahepatic gene profiling using microarrays in ethanol feed baboons has identified increased expression of 14 different annexin genes (including A1 and A2) that were not previously implicated in the progression of fibrosis in alcoholic liver disease [89]. Further, the intrahepatic transcriptome profile in alcoholism shares some similarity with LPS administration but in general is significantly different from other forms of liver disease [89,90]. Additionally, the hepatocyte transcript response to ethanol is significantly different compared to other hepatotoxins such as anticancer drugs [91].

Cluster analysis has allowed differentiation of alcoholic hepatitis from alcoholic steatosis. Genes known to be involved in alcohol injury such as alcohol dehydrogenases, acetaldehyde dehydrogenases, interleukin-8, S-adenosyl methionine synthetase, phosphatidylethanolamine N-transferase and several solute carriers have been shown to be differentially expressed in alcoholic hepatitis versus alcoholic steatosis. Many novel differentially expressed genes were identified, including claudins, osteopontin, CD209, selenoprotein and genes related to bile duct proliferation [89]. The most prominent categories of differentially expressed genes involved cell adhesion/extracellular matrix proteins, oxidative stress and coagulation that were also common to end-stage alcoholic liver disease. Genes associated with fibrosis/cell adhesion/ECM were the most prominent category in human advanced ALD, consistent with the fibrotic nature of ALD. However, these were not specific to alcohol, and have been reported in primary biliary cirrhosis and other forms of liver cirrhosis [76,81].

Non-alcoholic steatohepatitis (NASH) is the clinicopathological syndrome in non-alcoholic fatty liver disease (NAFLD) that has been most widely studied using gene array analysis of transcript expression. Studies have identified differentially expressed genes in end stage NASH cirrhosis compared to other disease states [92-94]. Decreased expression of genes associated with mitochondrial function and increased expression of genes associated with the acute phase response were observed [92]. The latter increases were speculated to be associated with insulin resistance, a feature of NAFLD [92]. Further differential expression of genes involved in lipid metabolism, extracellular matriz (ECM) remodelling, regeneration, apoptosis and detoxicification have all been observed in NASH following microarray analysis [93].

### Gene array analysis in human liver disease – biliary liver injury

Biliary liver injury has only been examined in a limited number of studies utilizing array analysis. The induction of Wnt pathway genes including Wnt13, Wnt5A, and Wnt12 was a striking and confirmed finding of gene array studies of primary biliary cirrhosis (PBC) [81]. Further novel PBC associated transcript expression included upregulation of Transcription initiation factor 250 kDa subunit (TAFII 250), PAX3/forkhead transcription factor and patched homolog (PTC). An unexpected but consistent feature of the gene array analysis of PBC was the repeated identification of differentially expressed *Drosophila *genes homologues (Wnt genes, hedgehog pathway, notch pathway) [81].

The only available data on primary sclerosing cholangitis (PSC) cirrhosis comes from a comparison to PBC cirrhosis [81]. Compared with PBC there were a far greater number of genes showing differential expression in PSC versus non diseased liver. These include genes associated with epithelial biology (Amphiregulin, Bullous pemphigoid antigen), inflammation (T-cell Secreted Protein P I-309, CTLA4), apoptosis related genes (Bcl-2 interacting killer, Bcl-x, Death associated protein 3) and intracellular kinases such as CDK7 and JAK1.

Biliary atresia (BA) has been studied by microarray analysis by comparing embryonic and perinatal forms of the disease [95]. Gene profiling clearly separated these two conditions. The most remarkable difference was in the expression of so-called regulatory genes. In Embryonic BA 45% of differentially expressed genes were in this category versus 15% in the perinatal form. Included in these genes were imprinting genes, genes associated RNA processing and cell cycle regulation that were not present in the perinatal form of BA.

### Gene array analysis in cultured cells

Experiments with cells in culture offer the advantage of a controlled environment in which to test specific hypotheses without influences at the level of the organ, organism or environment. Gene array technology has been used to characterise cells in culture in greater detail than previously possible. The techniques have been applied to study cellular differentiation and behaviour in response to toxins and various disease states. Despite the advances derived from this novel technology, limitations of the cellular models apply to these studies as well. For example, cellular function is altered by the culture conditions and may not represent those found *in vivo*.

### Gene array analysis in cultured cells – cellular differentiation

Gene expression array analysis has been applied to identify the multiple signals involved in cellular differentiation. Corticosteroids, hepatocyte growth factor (HGF), and epidermal growth factor are associated with mature histology in the organoid culture model [96]. Array analysis identified that these factors stimulate hepatocyte nuclear factor 4alpha (HNF-4alpha) expression in hepatocytes [96]. HNF-4alpha is a recognized liver-specific transcription factor and its effects on hepatic gene expression have also been studied. Almost half of the induced genes were metabolism genes many related to lipid metabolism which is frequently altered in liver disease [97]. Yamashita and colleagues determined target genes for hepatocyte differentiation and found that the Oct-3/4 transcription factor was upregulated while the early growth response-1 (EGR-1) transactivator was down-regulated [98]. Gap junctions are considered to play a central role in differentiation of hepatocytes. Connexin 32 (Cx32) is closely related to tight junctional proteins and can induce expression and function of tight junctions. To investigate the mechanisms of induction of tight junctions, cells transfected with Cx32 were analysed by cDNA microarray [99]. Expression of membrane-associated guanylate kinase with inverted orientation-1 (MAGI-1) was increased. MAGI-1 is known to be localized at adherens and tight junction regions. MAGI-1 was expressed in the apical-most regions at cell borders of Cx32 transfectants and co-localized with occludin, claudin-2, ZO-1, and F-actin [99].

### Gene array analysis in cultured cells – hepatocytes

Cultured primary hepatocytes have proven to be a valuable resource and extensively used research tool but questions remain regarding functional differences observed in these hepatocytes relative to the intact liver [97,98,100-103]. One study characterized cultured hepatocyte cell lines, primary hepatocytes in conventional monolayer or in sandwich culture, and liver slices based on mRNA expression profiles in comparison to gene expression in liver tissue [103]. Liver slices exhibited the strongest similarity to liver tissue regarding mRNA expression, whereas the two cell lines clustered together and were quite different from the whole liver. For selected cytochrome P450s the differences observed on the mRNA expression level there was a marked effect with the duration of culture. Expression patterns changed most rapidly soon after cell isolation and culture initiation and stabilized with time in culture [103]. A second study of cultured hepatocytes over time revealed time-dependent regulation of phase I and phase II metabolizing enzymes [100]. In general, cytochrome P450 mRNA expression was repressed, but expression of phase II metabolizing enzymes varied by class (upregulation of glucuronidation, down-regulation of sulfation). Progressive induction of several genes associated with the cellular cytoskeleton and extracellular matrix was observed in accord with physical changes in cell shape and connectivity associated with cellular adhesion [100].

Gene expression profiling has been utilised to define the molecular mechanism underlying epithelial non-parenchymal interactions in hepatocyte cocultures. Primary rat hepatocytes were cocultivated with closely related murine fibroblast cell types and revealed functional responses that correlated with fibroblast gene expression profiles. Two candidates playing an important role in functional differentiation were the cell surface protein neural cadherin (N-cadherin) and decorin [102].

### Gene array analysis in cultured cells – hepatic stellate cells

cDNA microarray was used to identify genes upregulated in activated hepatic stellate cells (HSCs) in culture [104,105]. In one study, a number of novel and previously recognised genes were identified including osteopontin (OPN) [104]. In another, a total of 835 differentially expressed genes were identified in an array comparison of activated and quiescent HSC. The differentially expressed genes included those involved in protein synthesis, cell-cycle regulation, apoptosis, and DNA damage response [105]. Functional expression of the telomerase catalytic subunit (human telomerase reverse transcriptase; hTERT) in human activated hepatic stellate cells (HSCs) rescues them from death with immortalization and maintains an activated HSC phenotype [106]. Senescent HSCs expressed reduced levels of extracellular matrix proteins, including collagens, tenascin, and fibronectin. Maintenance of telomere length represents an important survival factor for activated human HSCs [107]. Using this information, Schnabl and colleagues have created an immortalized human HSC line by infecting primary human HSCs with a retrovirus expressing hTERT [106]. Telomerase-positive HSCs did not undergo oncogenic transformation and exhibit morphologic and functional characteristics of activated HSCs. Microarray and RT-PCR analysis showed that mRNA expression patterns in telomerase-positive HSCs are very similar to those in activated human HSCs [106]. The immortalized HSC lines LX-1 and LX-2 were characterized by microarray analysis and determined to have a gene expression profile very similar to that of activated primary HSC [108]. These newly developed cell lines are proving to be valuable tools to study the biology of human HSCs.

### Gene array analysis in cultured cells – viral hepatitis

A particular challenge in the study of the effect of viruses on liver cells is the difficulty in infecting liver cells with virus. The studies described below have involved models in which cultured cells are infected with viral proteins or viral genome. Progress in this field has been rapid and most recently, a cellular model of HCV infection has been reported that is likely to stimulate further study [109,110].

To determine the oncogenic role(s) of HBx protein Hepatitis B virus (HBV) in the development of HCC, gene expression profiles in primary adult human hepatocytes and an HCC cell line (SK-Hep-1) ecotopically expressing HBx via an adenoviral system. Many genes including a subset of oncogenes (such as c-myc and c-myb) and tumour suppressor genes (such as APC, p53, WAF1 and WT1) were differentially expressed and cluster analysis showed distinctive gene expression profiles in the two cell types. HBx protein altered gene expression as an early event that favours hepatocyte proliferation that may contribute to liver carcinogenesis [111].

Interferon-alpha is currently the leading treatment for viral hepatitis. Several studies have used microarray analysis to identify the mechanisms by which interferon-alpha (IFN-alpha) acts on hepatocytes and the hepatitis C virus. IFN-alpha activated the multiple signal transducer and activator of transcription factors (STAT) 1, 2, 3, 5 in cultured hepatocytes [112]. Other up-regulated genes include a variety of antiviral and tumour suppressors/proapoptotic genes. Down-regulated genes include c-myc and c-Met and the HGF receptor [112]. In a second and comparable study, IFN-alpha antiviral efficacy was associated with 6–16 (G1P3) expression. Involvement of STAT3 in IFN-alpha signalling was confirmed [113]. Resistance to IFN-alpha antiviral activity may be mediated the hepatitis C viral protein, NS5A. To identify the mechanisms through which NS5A blocks interferon activity, gene expression profile was studied in IFN-treated Huh7 cells expressing NS5A. The strongest effect of NS5A on interferon response was observed for the OAS-p69 gene [114]. Another key response of hepatocytes to the HCV virus is cellular proliferation. Gene array studies identified upregulation of growth-related genes, in particular wnt-1 and its downstream target gene WISP [115]. In another study, CDK activity, hyperphosphorylation of Rb, and E2F activation was shown to be associated with hepatocyte proliferation induced by a full-length HCV clone [116].

### Gene array analysis in cultured cells – mechanisms of drug action

A number of studies have investigated the effects of toxins and therapeutic drugs in cultured liver cells using array techniques. Perhaps surprisingly, studies of the effects of alcohol on liver cell gene expression in vitro have not been reported. It has been proposed that transcription profiling can generate the information needed to assign a compound to a mode-of-toxicity class. Primary rat hepatocytes have been exposed to a variety of different hepatotoxins on the basis of their variety of hepatocellular effects [117,118]. A low-density DNA microarray containing 59 key genes was selected. All tested drugs generated a specific gene expression profile. Even with a relatively limited gene set, gene expression profiling allowed a certain degree of classification of compounds with similar hepatocellular toxicities such as cholestasis or necrosis. Clustering analysis linked the compounds known to cause hepatic steatosis. Drugs inducing necrosis and cholestasis clustered together and drugs classified as the CYP450 inducers formed individual clusters [119].

The pathways underlying ursodeoxycholic acid (UDCA) action were investigated by array analysis in primary rat hepatocytes [120]. In cells exposed to UDCA, >440 genes were modulated by >1.5-fold. Genes affected by UDCA included new regulatory molecules, such as Apaf-1. Other altered genes were directly involved in cell cycle (cyclin D1, cadherin 1, HMG-box containing protein 1) and apoptosis (prothymosin-alpha) events. The E2F-1/p53/Apaf-1 pathway appears to be targeted by UDCA [120].

Saturated fat plays a role in common debilitating diseases yet the molecular mechanisms of saturated fatty acid signalling in the liver are poorly defined. Hepatic gene expression analysis was undertaken in a human hepatocyte cell line after incubation with palmitate [121]. Altered transcription profiles were observed in a wide variety of genes, including genes involved in lipid and cholesterol transport, cholesterol catabolism, cell growth and proliferation, cell signalling, beta-oxidation, and oxidative stress response [121]. Streptozotocin (SZ) is known to exert toxic effects not only on pancreatic islet beta cells but also on other organs including the liver. Primary cultured hepatocytes were treated with SZ [122]. Gene expression analysis revealed up-regulation in cell proliferation/apoptosis related genes, and down-regulation of lipid metabolism related genes. These results indicate that many of hepatic alterations are direct effects of SZ rather than by the secondary effect of the hyperglycaemia or hypoinsulinemia [122].

Microarray analysis was conducted on isolated human hepatocytes to understand the mechanisms underlying the idiosyncratic toxicity induced by trovafloxacin [123]. The results clearly distinguished trovafloxacin from other marketed quinoline agents and identify unique gene changes induced by trovafloxacin that are involved in mitochondrial damage, RNA processing, transcription, and inflammation [123]. The mechanism(s) by which LPS stimulates cultured hepatocytes was studied by DNA microarray analysis. LPS modulates the selective expression of more than 80 genes and expressed sequence tags including members of relevant signalling pathways. NF-kappa B activation was reduced in TLR4-mutant or -null hepatocytes compared to control hepatocytes, and this defect was partially restored by adenoviral transduction of mouse TLR4. This study provided additional evidence that hepatocytes respond to LPS through a TLR4 response pathway [124].

### Gene arrays and animal models

Animal model studied include acute liver regeneration, drug toxicity, liver fibrosis, fatty liver, biliary obstruction, liver transplantation and carcinogenesis. Drug toxicity studies are numerous and include effects induced by clofibrate, PPAR alpha agonists, carbon tetrachloride, amiodarone, arsenic and methotrexate [117,125-134]. In one study, a novel cDNA library highly enriched for genes expressed under a variety of hepatotoxic conditions was created and used to develop a custom oligonucleotide library [135].

An expression signature for rat liver fibrosis was identified using a cDNA 14,814 gene microarray [136]. The "genetic fibrosis index" identified consisted of 95 genes (87 upregulated, 8 down-regulated). These included genes associated with cytoskeletal proteins, cell proliferation and protein synthesis. Bile obstruction in the mouse identified 3 sequential main biological processes. At day 1, enzymes involved in steroid metabolism were over expressed. This was followed by an increase in cell cycle/proliferation associated genes at day 7, occurring at a time of maximum cholangiocyte proliferation. From days 14–21 genes associated with the inflammatory response and matrix remodelling were identified. Similar temporal gene expression was identified in the model of acute liver regeneration. Steroid and lipid metabolism genes were down-regulated as early as 2 hours post hepatectomy whilst genes associated with cytoskeletal assembly and DNA synthesis became upregulated by 16 hours and remained elevated at the 40 hour time point at the peak of S phase.

Carcinogenic foci in experimental animals have been isolated and studied using gene array technology [137]. Approximately 8% of 2000 transcripts were differentially expressed in one study. These included genes with roles in signal transduction, detoxification and cytoskeletal assembly. Over 30 genes were identified as being dysregulated in these foci as well as in neonatal liver. Small for size liver allografts in rats showed upregulation of adhesion molecule, inflammatory mediators and apoptosis-associated genes together with a down regulation of energy metabolising genes.

Alcoholic liver disease has been studied in the chronic enterogastric ethanol infusion model in a mouse, a total of 12,422 genes were analysed [138]. Several cytochrome P450 genes were shown to be upregulated, whilst several genes involved in fatty acid metabolism (stear-a oil co-aid saturase 3-hydroxy-assile co-aid dehydrogenase) and fatty acid synthesase were down regulated. In contrast, genes associated with glutathione-s-transferase were markedly upregulated. Interestingly, a novel molecule intestinal factor was 50-fold down-regulated. It was postulated that alcohol may be affecting the healthy intestinal epithelium and down-regulation of this gene may be associated with permeability changes in the intestine associated with chronic alcohol ingestion.

### "Open system" analysis of transcriptomes

Although array analysis is the predominant technique for examining differential gene expression other techniques such as differential display, SAGE [139] and subtractive hybridisation [34] are useful complementary methods of examining transcriptomes.

#### Differential display

Differential display involves the use of non-stringent primer sets and PCR amplification to give a pool of products that are resolved on a polyacrylamide or PAGE gels [140-143]. The number of primer set combinations needed to screen a whole transcriptome varies but can be as many as 300 [142,143]. One of the benefits of differential display techniques is the ability to use small amounts of starting material as well as the ability to analyse multiple different samples [142,143]. Unfortunately, differential display is not a sensitive method of detecting rare RNA species [143]. Additionally, differential display has a high false positive rate of identifying differentially expressed genes [143]. Therefore, differential display is a labour intensive technique that requires additional methodology to confirm differential expression [140,141].

The application of mRNA differential display to liver disease has in general implicated previously uncharacterised or completely novel genes in liver pathobiology, especially in HCC pathogenesis. There are now in excess of 300 publications using differential display in liver disease. These studies include the identification of increased F-LANa expression in HCC [144]. F-LANa was a previously uncharacterised sequence associated with increased tumour cell growth [144]. Differential display has identified hepatic genes previously unsuspected as differentially expressed due to circadian transcription changes (including presenilin II) [145]. The application of differential display to evaluate the effect of iron overload on HepG2 cells has shown increased mRNA expression of semaphorin cd100 and aldose reductase and a decrease in apolipoprotein B100 mRNA expression [146]. Vitamin E administration blocks the increases in apolipoprotein B100 whilst H_2_O_2 _treatment increased only aldose reductase expression [146]. Therefore, it appears that iron affects both LPO-dependent and LPO-independent pathways. Further, studies have shown gene differential expression comparing peri-portal and peri-central hepatocytes identifying some of the molecular pathways responsible for the heterogenous nature of the hepatic lobule [147]. Further in HBV surface antigen transgenic mice, Pim-3, has been identified by differential display as a mediator of autonomous cell proliferation that is only expressed in HCC tissue[148]. The combined use of differential display and microarrays has identified sodium butyrate responsive genes such as CBl-2 and Mcl-1/EAT that appear to be involved in stimulating hepatocellular carcinoma cells into a normal phenotype[149]. This study demonstrates the utility of combining complementary methods of studying transcriptomes.

#### Serial analysis of gene expression

SAGE is a technique that relies on the fact that short nucleotide sequences of 9 to 11 bases contain enough information to identify a clone (known as a tag) [4,139,150]. SAGE uses a biotinolyated oligo dT primer to prepare cDNA that is cleaved with a 4-bp recognition site endonuclease [139]. The 3' end of the cDNA is then isolated using streptavidin and ligated to a linker that contains a type IIS restriction site [139]. The type IIS restriction endonuclease cleaves the cDNA giving a small 9 – 11 base sequence attached to the linker [139]. These multiple small sequences are concatenated together and sequenced [139]. Although SAGE provides both a quantitative and qualitative analysis of total gene expression it is not generally suited for widespread use given its reliance on intensive sequencing.

Serial analysis of gene expression has been applied to the pathobiology of human liver disease. Three studies have profiled normal human liver, chronic HCV liver and HCC tissue using SAGE [7,11,151]. In normal liver a total of 30 982 tags were identified that comprised 8 596 unique genes [7]. Tags that were expressed 10 or more times constituted 57.3% of the total tags but only 4.1% of the unique genes [7]. The breakdown of the unique tags showed that 21.8% were plasma proteins, 8.6% were cytoplasmic proteins, 4.8% were enzymes, 1.7% were protease inhibitors, 1.1% were complement components and 0.75% were coagulation factors [7]. Importantly, the function of at least 13.9% of the intrahepatic genes identified could not be determined [7]. The five most abundant transcripts identified in normal liver were albumin, apolipoprotein (Apo) A-I, Apo C-I, Apo C-II and ATPase 6/8 [7]. The abundance of selected SAGE transcripts correlated strongly with previously documented EST frequency in HepG2 cells (r^2 ^= 0.96) [7]. The same group in a related SAGE study of chronic HCV and HCC tissue, isolated 31 381 and 32 217 tags respectively [11]. This equated to 10 172 unique genes in HCV and 13 372 unique genes identified in HCC. Combing the SAGE results for normal, HCV and HCC tissue gave a total of 94 580 tags representing 24 464 unique genes [7,11]. Importantly, only 2 114 of these unique genes (8.6%) were expressed in all three SAGE libraries [7,11]. This suggests that these SAGE libraries were an incomplete representation of the hepatic transcriptome in normal and diseased states. In HCV cirrhosis the most abundant tags that were differentially expressed when compared to normal liver included; MHC class I, immunoglobulin κ chain, heat shock proteins, and DEAD/H box polypeptide 5 [11]. In HCC the genes that were increased included MHC class I, transmembrane protein BR1, glypican 3, DEAD/H box polypeptide 5 and CXCL-10 [11]. The 116 unique genes identified were differentially expressed in HCV compared to HCC tissue [11]. The results from Yamashita et. al. [7,11] contrast with those of Kondoh et. al. [151], who used a modified SAGE method to generate 50 515 tags from HCC tissue and 50 472 tags from cirrhotic tissue (4 out of 5 of these patients had HCV cirrhosis) representing 20 534 and 15 163 unique clones respectively. However, Kondoh et. al. found only eight known genes that differed between HCC and the cirrhotic tissue (galectin4, UDP-glucuronosyltransferase, ribosomal phosphoprotein P0, dek, IGFBP-1, vitronectin, retinoic acid induced gene E and CYP IIIA4) [151]. Further, Kondoh et. al. could only confirm the differential expression of a single gene, CYP IIIA4, by Northern blot analysis [151]. The results of all three SAGE studies suggest that the hepatic transcriptome increases in complexity with disease. The explanation for the marked difference in the extent of gene expression between HCC and cirrhotic tissue (predominantly HCV cirrhosis) in two of these studies is unclear. SAGE analysis has also been used to identify biomarkers of non-parenchymal cell populations. A comparison of over 70 000 transcripts from liver sinusoidal endothelial cells (LSEC) with and without CCl_4 _administration has identified multiple genes including Cdkn1a and Irf1 upregulated with injury and Stab2 a marker of LSEC [152].

### Subtractive hybridisation

Subtractive hybridisation is a method of enriching for differentially expressed genes in one gene pool compared to another [34]. The essential feature of this method is that one gene pool is labelled or tagged enabling the separation of unique transcripts from the tester (also called tracer) gene pool following hybridisation to an excess of driver cDNA [34]. The starting material is often limited and amplification is performed prior to subtraction using a number of techniques such as poly(A) RT-PCR, Eberwine amplification and SMART cDNA synthesis (Clontech, CA, USA) [34]. The technique classically uses a biotinylated driver enabling common sequence in the driver and tester gene pools to be removed using streptavidin precipitation [34]. Multiple rounds of subtraction can be performed giving a tester gene pool enriched for differentially expressed genes [34]. A number of techniques related to subtractive hybridisation include representational differences analysis (RDA) and suppression subtractive hybridisation (SSH). Representational differences analysis uses PCR amplification of the unique transcripts in the cDNA pool [153,154]. This can give a greater than 10^6 ^fold enrichment of differentially expressed cDNA in three rounds of amplification [153,154]. RDA has been utilised to find unique genomic DNA mutations as well comparing mRNA transcript pools [153,154]. SSH utilises PCR amplification (like RDA) combined with the suppression effect of PCR [155,156]. The suppression effect is mediated by the incorporation of long inverted terminal repeats which, when attached to the ends of DNA fragments, form stable panhandle-like loop structures that are favoured over the shorter PCR primers [155,156]. Therefore, undesirable sequences are not amplified in the PCR.

All subtractive hybridisation approaches simply enrich for differentially expressed genes. Individual gene differential expression needs to be confirmed by supplemental methodology. The supplemental methodology used varies but includes northern blot analysis and PCR based methods. Northern blot analysis, although an accurate method of quantification, that additionally gives information about mRNA transcript size, is limited by being labour intensive and lacks the throughput necessary to match the subtractive hybridisation approach. Additionally, Northern blot analysis is restricted to moderate to high abundance transcripts. PCR based methods are now the preferred means of confirming differential gene expression. Real-time quantitative RT-PCR is a rapid and effective means of confirming gene differential expression. The multiple methods of performing real-time RT-PCR include the use of FRET probes, molecular beacons and the use of the double strand intercalating flurophore Sybr Green 1 [157-159]. Real-time RT-PCR has the additional benefit of being able to confirm differential expression of lowly expressed mRNA transcripts [160]. Indeed the use of real-time RT-PCR is now used not just to confirm differential gene expression following subtractive hybridisation but also to confirm differential gene expression following cDNA array analysis, differential display and SAGE.

Subtractive hybridisation has been used to investigate the pathobiology of human liver disease. There are now in excess of twenty papers that utilise subtractive hybridisation to investigate liver injury. The SSH variation, described above, is now the most commonly utilised subtractive hybridisation method [156,161]. Further, SSH unlike mRNA differential display appears to have a greater yield in terms of the number of genes identified that are both known and novel in an individual experiment. The identification of gankyrin in HCC using SSH is a significant finding as this oncoprotein, with ankyrin repeats, has been demonstrated to increase anchorage-independent growth and tumorigenicity in NIH/3T3 cells [162]. Gankyrin appears to increase the phosphorylation of the retinoblastoma gene with activation of the E2F-1 transcription factor [162]. Studies of iron overload have previously demonstrated iron overload in knockout mice lacking the Usf-2 transcription factor in a pattern similar to the HFE -/- mouse, the murine model of genetic haemachromatosis [163]. Comparison of intrahepatic gene expression in Usf-2-/- and Usf-2+/+ mice by SSH showed a marked down-regulation of the mRNA encoding hepcidin (also known as liver expressed antimicrobial peptide) [163]. Concurrently, Pigeon et. al. also identified increased hepcidin expression using SSH in carbonyl treated mice that have iron overload [164]. Therefore, hepcidin appears to act in conjunction with HFE to regulate intestinal iron absorption and iron storage in macrophages although the exact mechanism is still unknown [163,164]. The study of hepatitis C infected livers using SSH confirms a Th1 associated immune profile with CXCL-10, IFN regulated MxA, IFN induced p44, and IFN induced p56 (IFI-56 K) all increased greater than 4 fold in chronic HCV infection [165]. The identification of increased CXCL-10 in HCV infection has now been demonstrated using both array analysis and SSH [165,166]. Further, a study of PBC using SSH identified a total of 71 differentially expressed sequences with 62 being known genes and the remaining 9 clones homologous to EST sequences [167]. Interestingly, two of the sequences enriched for in PBC included Wnt13 and notch2 suggesting an involvement of these highly conserved *Drosophila *pathways in PBC pathobiology [167]. Unfortunately, many of the studies utilising SSH do not utilise supplemental methodology to confirm differential gene expression.

Our own work with SSH utilized this technique as a method of bio-discovery rather than a means of profiling transcriptomes. By comparing human HCV, AIH, PBC and non-diseased liver in multiple paired comparisons we were able to uncover completely novel sequences involved in the pathogenesis of these human liver diseases [82]. The reliance on sequencing clones helped us identify previously unrecognised spliced variants of the gene RERE in HCV cirrhosis [82]. Further our results identified genes such GP-39 and 2,5-oligoadenylate synthetase that had been identified previously by other techniques [82]. Additionally, we extensively used real-time RT-PCR to validate the observed gene expression [82].

## Conclusion

The study of transcriptomes using functional genomic methods is beginning to unravel the complexities of the human gene expression. The use of functional genomics methods, led by gene array analysis, has significantly advanced our understanding of organ and cell specific transcriptomes. Hepatic specific transcriptome analysis has addressed important aspects of viral hepatitis infection, xenobiotic metabolism, alcohol effects and liver transplantation. However, there have been comparatively few studies of the normal liver transcriptome. Questions about hepatic transcriptome differences due to factors such as diet, age, gender and ethnicity remain unanswered. Additionally, the relationship of the hepatic transcriptome to the proteome has demonstrated that a significant proportion of proteins are not regulated by the expression of mRNA transcripts. Further, most of the studies to-date "sample" a portion of the transcriptome rather than profiling entire transcriptomes.

Complementary methodologies including SAGE, DD and SSH demonstrate the benefits and deficiencies of gene array analysis of transcriptomes. The application of multiple methods to study transcriptomes enables profiling as well as bio-discovery of known and novel sequences. Further, the use of supplemental methodology to confirm observed differential gene expression is both necessary and increasingly widespread. Finally, a discussion of transcriptome analysis would be incomplete without recognition of the unique role of proteomic methodologies that provide researchers with yet another option of profiling the expression of the genome. Indeed the distinction between the transcriptome and proteome is becoming blurred as we are now beginning to focus on the "phenome" [168,169].

In conclusion, whole transcriptome analysis is a means of examining organ and cell pathobiology. The caveats are many but the potential advances in understanding liver disease are clearly immense. In contrast to a reductionist approach, examination of the entire transcript milieu will help us define gene relationships and patterns of expression that define disease. The promise heralded by the sequencing of the genome is being realized!

## Competing interests

The authors declare that they have no competing interests.

## Authors' contributions

NAS was responsible for preparing the manuscript. DS was responsible for the section on alcoholic liver disease. PSH was responsible for the section on liver specific cell populations. MDG provided expert assessment and critical appraisal. GWMcC was the senior author. All authors have read and approved the final manuscript.
